# The immune responses of *Oreochromis niloticus* against *Prohemistomum vivax* encysted metacercariae infection with the evaluation of different biomarkers stressors

**DOI:** 10.1038/s41598-023-38809-z

**Published:** 2023-07-23

**Authors:** Nehal A. Younis, Hasnaa Thabit, Salma I. El-Samannoudy, Marwa M. Attia

**Affiliations:** 1 Department of Aquatic Animal Medicine and Management, Faculty of Veterinary Medicine, Cairo University, Giza, 12211 Egypt; 2 Department of Zoology and Entomology, Faculty of Science, Assiut University, PO 71526, Assiut, Egypt; 3 Physiology Department, Faculty of Veterinary Medicine, Cairo University, Giza, 12211 Egypt; 4 Department of Parasitology, Faculty of Veterinary Medicine, Cairo University, Giza, 12211 Egypt

**Keywords:** Immunology, Physiology, Zoology

## Abstract

This study aimed at evaluating the immunological status of *Oreochromis niloticus* (*O. niloticus*); so, a total of 120 *O. niloticus* were collected from different farms located in Kafr El-Sheikh Governorate in Egypt during the period from January 2021 to January 2022. The fish were surveyed for commonly encysted metacercariae present in different organs such as gills, spleen, liver, kidney, and muscles. The collected encysted metacercariae were of the family Cyathocotylidae (*Prohemistomum vivax*) with a prevalence of 25%. Different cell-mediated immune responses such as *Major histocompatibility class II alpha* (*MHC-IIα*), *Toll-like receptor 7* (*TLR-7*),* Interleukin* (*IL-8*), and* Clusters of differentiation 4* (*CD4*) were assessed in different organs such as gills, spleen, liver, kidney, and muscles which revealed an elevation in different genes in infected organs as a reaction from the body against parasitic infection. In addition, the liver enzymes; aspartate aminotransferase (AST), and alanine aminotransferase (ALT), were assessed in the serum of *O. niloticus* as well as blood glucose, cortisol levels, and lysozyme activity were estimated to record higher levels in the infected fish in comparison with the control non-infected ones.

## Introduction

*Oreochromis niloticus* (*O. niloticus*) plays a significant commercial role in Egypt, yet wild and cultured tilapia are facing a severe loss because of the attack of various ectoparasites, helminths, and protozoa which cause various degrees of diseases in such fish^1^. *Oreochromis niloticus* is the primary freshwater fish species found in Egypt’s Nile River (Nile tilapia). For all Egyptians, it is one of the most common and inexpensive fish. Due to their somatic illness resistance and low respiratory demands, they can withstand harsh environments, including low oxygen and high ammonia levels^2^. Diseases caused by parasites in fish are common worldwide, particularly in the tropics^3^. One of the most common and effective ways that living things survive is through parasitism^4^. Therefore, fish parasitology is a crucial tool in research on aquatic health for developing a control strategy that requires a solid grasp of parasite biology^5,6^. The health of people and domesticated animals as well as the safety of food, and the aquaculture world are all threatened by fish-borne trematodes, especially those that are zoonotic in origin (fish-borne zoonotic trematodes, or “FZT”)^7–9^.

Second intermediate hosts can include a wide variety of creatures, such as snails, bivalves, aquatic insect larvae, crustaceans, frogs, fish, and reptiles which infect the fish with encysted metacercariae. More importantly, many fish parasites, especially trematodes, are major zoonotic agents that can seriously harm human health by causing diarrhea and abdominal pain. The major way that humans become infected is through eating raw or inadequately cooked fish that contains encysted metacercariae. Recent additions to the list of emerging infectious illnesses include fish-borne zoonotic trematodes, according to the World Health Organization and the United Nations Food Agriculture Organization^10^.

Cytokines are released by immune cells that have been triggered in response to a variety of pathogens, such as parasitic, bacterial, or viral components^11^. When they attach to the appropriate receptors, they can modify immune responses in an autocrine or paracrine manner. *Interferons* (IFNs), *interleukins* (ILs), and *tumor necrosis factors* (TNFs) are all considered stimulating factors, while chemokines are different types of cytokines that are produced by macrophages, lymphocytes, granulocytes, mast cells, and epithelial cells^12–14^. *IL-1*, *IL-6,* and *TNF-α* are all necessary for pathogen elimination and the recruitment of macrophages, neutrophils, and lymphocytes to the diseased tissues in innate immunity^15^.

The *major histocompatibility complex* (*MHC*) genes play a critical role in adaptive immunity. Class I and class II genes, which make up the two main antigen-presenting groups of *MHC* molecules, have different functions, structures, and patterns of expression. The primary role of *MHC class II* genes is to encode cell-surface glycoproteins that bind foreign peptides to present self- and non-self peptides to the T-cell receptor of *CD-4*, which in turn triggers a particular immune response against the pathogen from which the peptides are derived. Previous research has demonstrated that the immune system’s cells, including antigen-presenting cells, are primarily found in the kidney, gut, gills, and spleen, where *MHC* genes are primarily expressed^15^.

One of the most important elements of the fish’s non-specific immune system is lysozyme, a polypeptide with a molecular weight of 14–18 kDa. Many different vertebrates, including freshwater and marine fish, contain the enzymes. Following the destruction of the outer wall by complement and other enzymes, it acts directly on different pathogens such as Gram-positive and negative bacteria. White blood cells, particularly neutrophils, and monocytes, as well as leukocyte-rich organs such as the kidney, spleen, skin mucus, gills, and digestive system, release lysozyme, which has antibacterial properties^15^.

*Oreochromis niloticus* experiences distinct reactions in response to different stressors in case of infection, which increase the levels of plasma cortisol, the primary hormone that activates glucose, and consequently glucose levels. Fish respond to stress by elevating blood levels of glucose and cortisol. Chronic stressors cause the release of cortisol. Stress is indicated by elevated plasma cortisol and glucose levels^16^.

Therefore, this work aims to identify the relationship between the different cell-mediated immune responses of Nile Tilapia (*O. niloticus*) infected with encysted metacercariae, liver enzymes analyses, lysozyme activity as well as blood glucose and cortisol levels of the infected fish as a reaction from the body against parasitic infection.

## Materials and methods

### Collection of fish

One hundred and twenty Nile Tilapia (*O. niloticus*) were sampled from various farms located in Kafr El-Sheikh Governorate (31.3° N 30.93° E) in Egypt between January 2021 and January 2022. The examined fish ranged in total length from 8 to 16 cm and weight from 50 to 85 g.

These fish were transferred alive for examination of encysted metacercariae (EMC) in different organs. The fish were taken to the lab while still alive to be examined for any parasites and to study the response of the immune system^17^. Fish were brought to the lab alive in aquariums filled with recirculating water and an oxygen source.

### Parasitological examination

Each fish was euthanized using an overdose of commercial clove oil (Ectyo-colve®, France using at a concentration of 1239 ppm and a ratio of 1:4 to anhydrous ethanol); then the fish was necropsied and the mucous surrounding the skin, gills; fins, muscles around the head, muscles around the abdomen, liver, kidney, spleen, intestine, and eyes were examined under a light microscope (Olympus CX41 microscope; Japan). The muscles were compressed between two slides and examined under a stereoscopic microscope for the presence of any encysted metacercaria (EMC)^18–20^.

### Quantitive real-time polymerase chain reaction (qRT-PCR)

#### Sampling

Under sanitary conditions, samples from the gills, spleen, liver, kidney, and muscles infected with encysted metacercariae (EMC) were collected. Five non-infected control fish were sampled in the same way.

#### RNA isolation

The sampled fish (five infected fish) selected for expression analysis harbored only encysted metacercariae (EMC). The mRNA from 100 mg of the examined samples was isolated using a total RNA isolation kit (Ambion, Applied Biosystems), following the manufacturer’s instructions. The tissues were homogenized in a FastPrep-24 homogenizer using Lysing Matrix D tubes (MP Biomedicals)^21^. Using Nanodrop, the generated mRNA purity and quantity were evaluated (Thermo Scientific). Using the instructions provided in the manufacturer’s procedure, the High-Capacity cDNA Archive Kit (Applied Biosystems) reverse-transcribed the DNaseI-treated mRNA^22^.

#### The protocol of the *q*-Rt-PCR

According to the sequences particularly for *O. niloticus* that were deposited in the GenBank and listed in Table 1^23,24^, PCR primers for various studied genes were created. Following the procedures of Akbari et al.^25^, for cDNA synthesis 2 μL of RNA extract was added to a hexamer primer solution to create cDNA; the mixture was then immediately put on ice for at least one minute after being incubated at 65 °C for five minutes in a thermal cycler. Ten microliters of a first standard reaction (2×) containing reverse transcriptase (2 μL), 10 mM MgCl_2_, and 1 mM dNTPs were incubated at 25 °C for 10 min, the PCR programming cycle were followed according to Attia et al.^26^.

**Table 1:** Primers used in cell-mediated immune responses analyses.

Primers	Sequence (5ʹ–3ʹ)	Accession number
MHC-II α	F-TGGCCCTGACTGAACCACTG	NM_001282888.1
	R-TCAGACCCACGCCACAGAAC	
TLR-7	F-TCAGCAGGGTGAGAGCATAC	XM_005477981.1
	R-ACATATCCCAGCCGTAGAGG	
IL-8	F-GCACTGCCGCTGCATTAAG	NM_001279704.1
	R-GCAGTGGGAGTTGGGAAGAA	
CD-4	F-AAGAAACAGATGCGGGAGAGT	XM_005455473.3
	R-AGCAGAGGGAACGACAGAGAC	
β-actin	F-GGCTACTCCTTCACCACCACAG	KJ126772.1
	R-GGGCAACGGAACCTCTCATT	

### Determination of liver enzymes, glucose, and cortisol levels

Blood samples from five infected and five non-infected fish were subjected to biochemical analysis. Fish given benzocaine (50 mg/L) for anesthesia were used for blood collection from caudal veins and blood was drawn with and without an anticoagulant. To obtain serum, blood was allowed to coagulate at 4 °C, centrifuged for 15 min at 1500 rpm, and then maintained frozen at − 20 °C for biochemical examination. Using commercial kits (Spectrum-diagnostics, Egypt) and a JASCO V-730 spectrophotometer (JASCO, Tokyo, Japan), glucose, Alanine aminotransferase (ALT), and aspartate aminotransferase (AST) levels were calculated. A commercially available cortisol kit was used to test blood cortisol levels by radioimmunoassay, and a liquid scintillation counter was used to measure radioactivity^27^.

### Assessment of serum lysozyme activity

Based on the lysis of* Micrococcus lysodeikticus* (Sigma, USA), serum lysozyme activity was determined following Dotta et al*.*^28^, with some changes. 0.2 mg/mL PBS, pH 6.2, and 0.75 mL of *M. lysodeikticus* solution were combined with 0.25 mL of serum. The reaction was conducted at room temperature, and from 0 to 20 min, the absorbance at 450 nm was determined (Photometer, BM Co. Germany). Lyophilized chicken egg-white lysozyme was used to create a calibration curve that was used to determine the serum lysozyme concentrations (Sigma, USA)^29^.

### Statistical analysis

All data were presented as range (mean ± standard error) using a Independent Sample *T*-test which was performed to compare the presence of immunological genes in different tissues between infected and non-infected groups.

### Ethics approval, and consent to participate

This study was approved by the Institutional Animal Care and Use Committee, Faculty of Medicine, Assiut University, Egypt, (IRB No. 17300859).

### Compliance with relevant guidelines and regulations

Clinical examination, dissection, sampling, sample processing, microscopical examination, physiological and immunological analyses were carried out in accordance with relevant guidelines and regulations supported by relevant references throughout the manuscript materials and methods section.

### Compliance with ARRIVE guidelines

The current study was carried out in compliance with the ARRIVE guidelines when relevant methods were applied.

## Results

**Figure 1:**
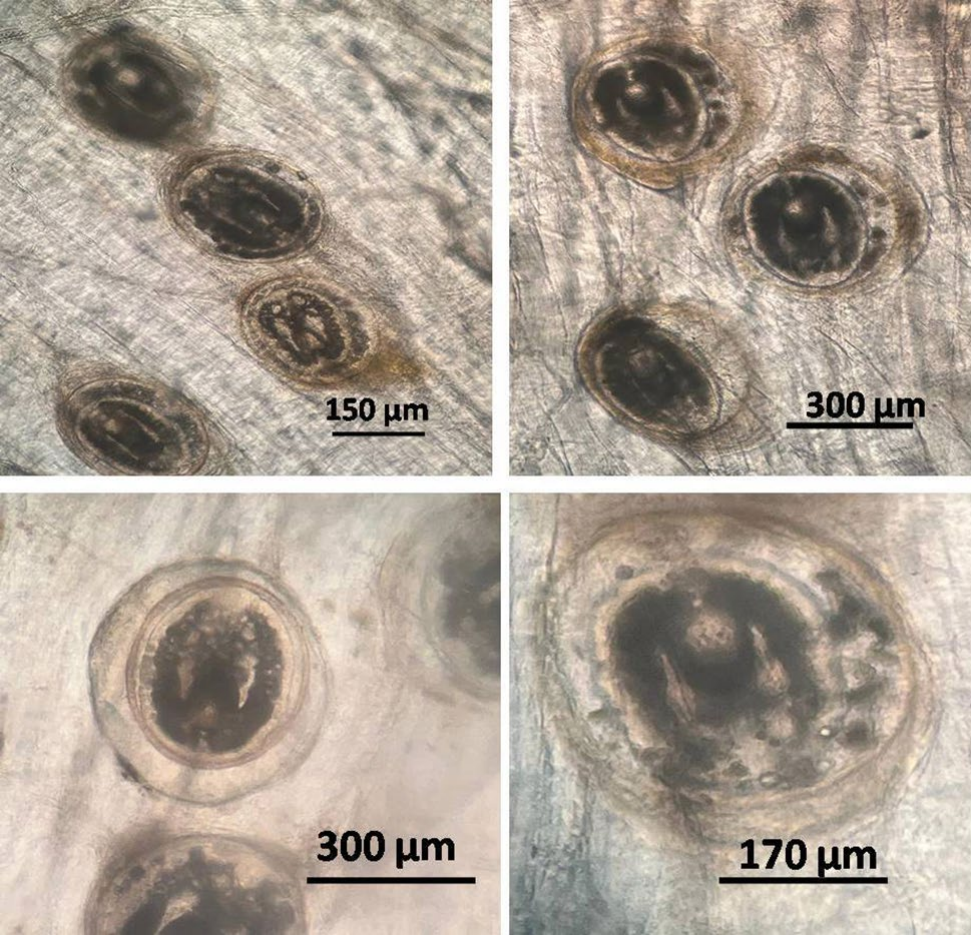
*Prohemistomum vivax* encysted metacercariae collected from infected muscles.

The collected encysted metacercariae were of the family Cyathocotylidae *(Prohemistomum vivax*). *Prohemistomum vivax* encysted metacercariae were discovered in different fish organs (muscles, skin, eyes, intestine, liver, kidney, and gills) of infected *O. niloticus*, which is oval to rounded in its shape, the *P. vivax* measured 335–385 (378 ± 5.6) μm in length and 315–320 (310 ± 5.5) μm in breadth. The cysts had a firm inner wall and a brittle double wall on their outer layer, which was brownish. The cyst was surrounded by two lobulated sacs on either side (Fig. 1).

The encysted metacercariae of *Prohemistomum vivax* were recorded during one year with 25% (30/120 fish) of the investigated samples having intensity of infection with 1–10 cysts per microscopic field.

**Figure 2:**
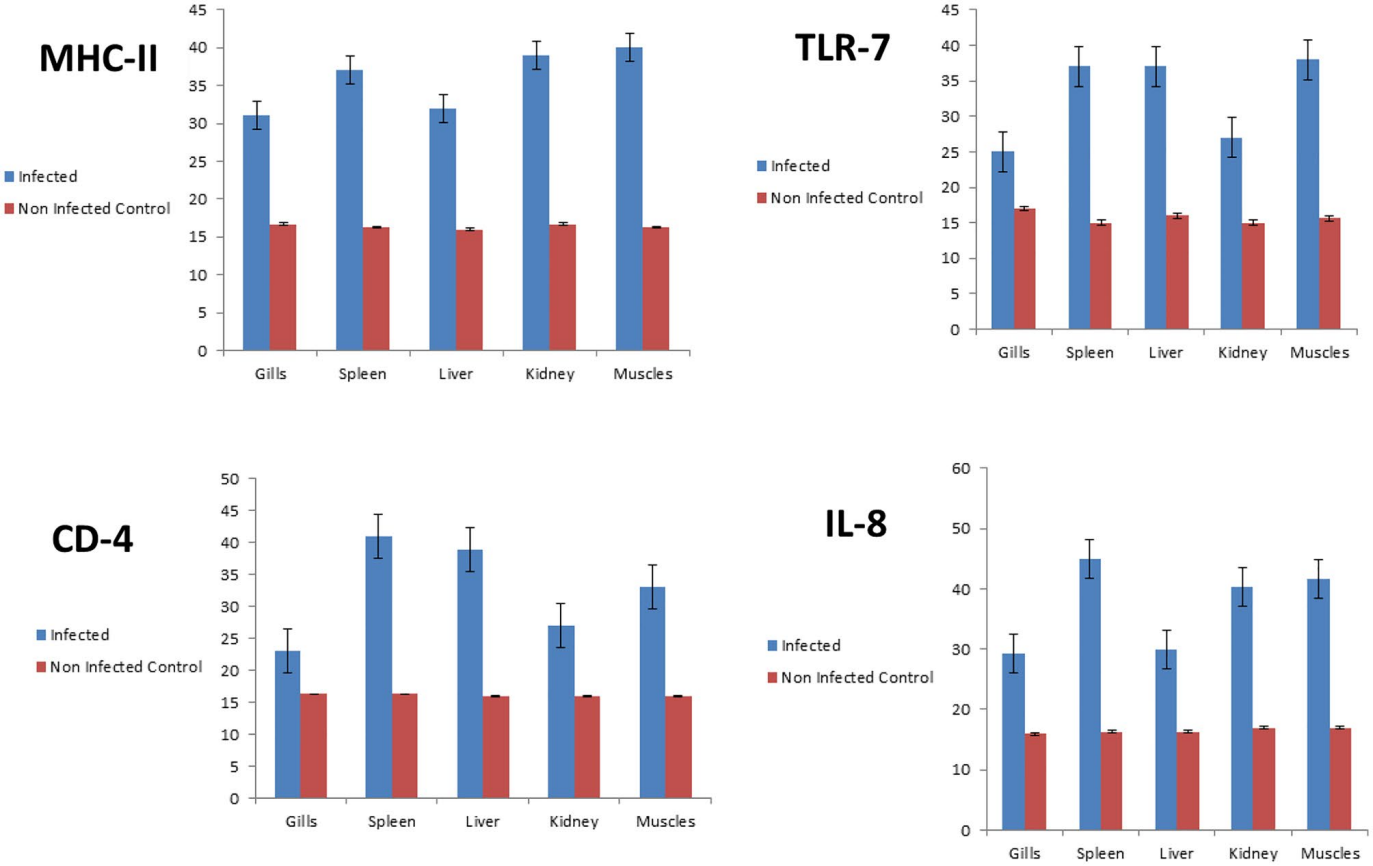
Transcription analyses of different organs with different genes.

The transcriprit of *MHC-IIα* were upregulated in different fish organs than in the control non-infected fish, the muscles, kidney, and spleen were the highest upregulation than the liver and gills (Fig. 2). Dealing with *TLR-7* gene was upregulated in different organs as the muscles, spleen and liver were higher in upregulation than the kidney and gills (Fig. 2). The *CD4* gene was upregulated in the spleen followed by the liver and muscles which higher than the kidney and gills (Fig. 2). The *IL-8* gene was upregulated in the spleen followed by the muscles and kidney which higher than the liver and gills (Fig. 2).

The mean value of lysozyme level in the serum of examined *O. niloticus* infected with EMC (*P. vivax*) was 155–159 (156.66 ± 1.2) μg/mL, while in the control non-infected fish it was 85–87 (86 ± 0.6) μg/mL.

In comparison to the control non-infected fish, the infected fish had significantly higher levels of AST, ALT, cortisol, and glucose Table 2.

**Table 2:** Liver and kidney function tests as well as stress factor; cortisol and glucose levels in the infected and non-infected fish.

Fish	Test			
	AST (IU/L)	ALT (IU/L)	Cortisol (ng/mL)	Glucose (mg/dL)
Infected	110–120 (114 ± 3.1)	43–40 (39 ± 2.3)	45–76 (63 ± 9.3)	114–132 (122 ± 5.3)
Non-infected	44–48 (46 ± 1.2)	18–21 (19.7 ± 0.9)	28–33 (30 ± 1.5)	45–66 (55.7 ± 6.1)
*T*-test	20.8	7.9	3.5	8.2
*P* value	0.000	0.001	0.025	0.001

## Discussion

In this study, several encysted metacercariae in different organs were recorded and isolated for identification of the species which found it belonged to the Cyathocotilade family (*P. vivax*). We found a significant relationship between different criteria as parasites, and immunogenic relationships all of which had retard effects on the condition of the tissues.

The mRNA expression levels of cytokines and inflammatory molecules, including *Toll-like receptors* (*TLRs*) and cell signaling molecules involved in innate fish immune responses have been extensively studied^30^. In teleosts, the head kidney serves as the major lymphoid tissue compared to the bone marrow in vertebrates^31^. Since *TLRs* constitute a significant subset of pattern recognition receptors (PRRs), activating them can cause macrophages to create inflammatory cytokines, which in turn trigger the synthesis of chemokines derived from macrophages and increase the phagocytic activity of macrophages^32,33^. Leukocyte mobility is aided by chemokines in response to pathogenic exposure and controls immunological responses in this way. Several fish species can develop adaptive immune responses in their systemic organs following sub-lethal infection^34,35^. Some soluble and membrane-bound proteins work with the complement system, a crucial component of the innate immune system, to destroy pathogens^36,37^. Microorganisms and antibody-antigen (Ag-Ig) complexes can both directly activate complement-mediated death^38^.

In this study, we evaluate several immunological genes in different infected organs: gills, muscles, spleen, liver, and kidney. Fish gills are frequently infected with parasites, yet these areas serve as barriers to other parasites by secreting mucus, which reduces the parasite load. In response to parasitic protozoan infection, cultured *O. niloticus* can respond immunologically and upregulate the expression of several genes^39^.

By secreting various immunoglobulins, lectins, lysozymes, and complement C-reactive protein, mucus serves as the first immunological barrier of the body to protect against various infections, according to Zhu et al.^12^ and Jones^39^. These results mean that in the skin, gills, muscles, and liver the upregulation was higher due to the presence of mucous and macrophages as reported by Zhu et al.^12^, which explains different cytokines which secrete several products from mast cells, macrophages, and lymphocytes. These products were interferons, interleukins, and tumor necrosis factors.

Due to their critical function in immune protection and illness management, *MHC* genes have garnered a great deal of research. One of the most crucial characteristics of *MHC* genes is high polymorphism, which serves as the foundation for searching for gene markers linked to disease resistance. Class A and B genes in the *MHC- II* family, which encode the a and b chains, are significant members of the *MHC* family.

They both combine to produce class II heterodimers, which result in a useful protein that is present on the cell surface. They are crucial in bringing extracellular pathogens’ foreign peptides together and presenting them to helper T cells.

Many pathological and ecotoxicological studies have used hematological indicators as biomarkers of fish health status. In the current study, two groups, i.e. non-infected fish and infected ones, were examined for certain physiological factors connected to the liver of Nile Tilapia, including alanine aminotransferase, which elevated in the infected fish than the control non-infected ones as a result of an infection in the kidney and liver with malfunction. Other investigations have shown that these enzymes are elevated in Nile Tilapia, *Oreochromis niloticus*, in response to parasite pollutions like external protozoa and monogenetic trematodes^40^, which are in agreement with our findings. This can be brought on by the liver producing more enzymes or by hepatic cells being damaged^41^. An essential enzyme in the metabolism of amino acids is aspartate aminotransferase, which catalyzes the reversible transfer of an a-amino group between aspartate and glutamate.

The innate immune system’s key defense molecule, lysozyme, is crucial for mediating protection against microbial invasion. It is a leucocytic-derived mucolytic enzyme, so the lysozyme is greatly elevated than normal against severe infection with EMC^42,43^.

## Conclusion

From the previous explanation of the infection of *O. niloticus* with the high prevalence of the parasitic disease; these parasites may affect human health. This destruction is present in the fish tissues and appeared in lysozyme activity and kidney and liver function. So, the water in the aquarium must be changed and cleaned regularly, and the wildlife and snails in the fish ponds must be cleaned regularly. Different rules in sanitation and health condition for ponds must be taken into consideration.

## Data Availability

All data generated or analyzed during this study are included in this article.
